# Investigations on the Processing of Ceramic Filled Inks for 3D InkJet Printing

**DOI:** 10.3390/ma13112587

**Published:** 2020-06-05

**Authors:** Dennis Graf, Afnan Qazzazie, Thomas Hanemann

**Affiliations:** 1 Laboratory for Materials Processing, University of Freiburg, D-79110 Freiburg, Germany; afnan.qazzazie@imtek.uni-freiburg.de (A.Q.); thomas.hanemann@kit.edu (T.H.); 2 Institute for Applied Materials, Karlsruhe Institute of Technology, D-76344 Eggenstein-Leopoldshafen, Germany

**Keywords:** composites, photopolymers, 3D inkjet printing, ceramic inks

## Abstract

3D inkjet printing is moving from a technology of rapid prototyping to rapid manufacturing. The introduction of ultraviolet curable composites filled with functional ceramics could expand the possibilities of this technology. In this work, a simple and scalable process was investigated as a template for the production of inkjet printable functional ceramics. Pyrogenic alumina particles with an average size of 13 nm, 35 nm and 100 nm were used as fillers in an acrylate mixture. The physical coating of the ceramics with 2-[2-(2-methoxyethoxy)ethoxy] acetic acid results in a low-viscosity dispersion with a ceramic content of up to 2 vol%, Newtonian behavior and surface tension within the limits allowed for inkjet printing. The material has sufficient stability for printing tensile specimens. Tensile tests have shown that modulus of elasticity, tensile strength and toughness can be kept constant despite the light scatter caused by the particles. The final production steps could be reduced to grinding and drying of the powders, their resuspension in the organic matrix and inkjet printing. The process can be used in an industrial-scale production of materials for abrasion-resistant components with adapted tribology.

## 1. Introduction

Additive manufacturing is changing the landscape of production in areas like automotive, aerospace and medical devices [1,2,3,4,5]. Techniques such as fused deposition modeling, selective laser sintering, stereolithography, electron beam melting and 3D printing are increasingly used [4,5]. However, no single technology can meet all manufacturing requirements. Rather, the technology must be carefully selected according to the application [6,7,8,9]. This leads to the development of further new and sophisticated production methods. For example, when it comes to printing ever smaller structures [10,11] or simplifying the process by using physical conditions to meet economic requirements [12,13]. 3D inkjet printing differs from the above-mentioned production methods in that it allows several materials to be deposited in one process step, which could enable the production of fully functional and complete components in one printing process [14,15,16]. Layer by layer, a low-viscosity material is usually dispensed from a drop-on-demand print head via piezoelectric actuation. Therefore, the viscosity of the material at printing temperature is usually limited to a range between 10 mPa·s and 100 mPa·s. The surface tension should be in the range of 25–50 dyn/cm. Due to the small nozzle diameters of 30 µm to 50 µm, an accuracy of about 30 µm can be achieved [17,18]. The current range of commercial materials includes polymers with varying degrees of elasticity, strength and toughness. Conductive printing inks are also increasingly being investigated for use in printed 3D electronics [19,20,21,22,23]. Although the composition of most materials is confidential, it is likely that a large proportion are highly crosslinkable mixtures of mono- and polyfunctional monomers and oligomers with acrylate and methacrylate groups [24,25]. These materials are ultraviolet (UV)-curable and therefore have the advantage of immediate chemical solidification and low vapor emission [18,26]. They react in a photoinitiated bulk polymerization in which light-sensitive additives, usually α-cleavable molecules, e.g., acylphosphine oxides, and hydrogen-removing compounds, e.g., aromatic ketones with a radical precursor, initiate chain growth [18,27].

To further increase the potential of 3D inkjet printing, the technology is moving from rapid prototyping to rapid manufacturing [14,15]. This creates a demand for components with suitable mechanical properties. Among other things, parts such as bearings must be resistant to abrasion and have an adapted coefficient of friction. Even low contents of ceramics embedded in photopolymers can improve the mechanical properties of coatings [28]. This knowledge can be transferred to the formulation of 3D-printable materials. However, the addition of ceramic fillers strongly influences the curing behavior of nanocomposites. A reduction of the photopolymerization rate and double bond conversion can be a consequence and could weaken the positive effects of nanofillers on the mechanical properties. The reasons for this are, firstly, the light scattering at the particles [29] and secondly, an increase in the viscosity of the nanocomposite restricts the mobility of the acrylic radicals [30]. Third, the nanoparticles act as additional crosslinkers and contribute to early vitrification [31]. In order to keep the curing speed constant and achieve a high conversion of the educts, a low nanoparticle content is, therefore, advantageous [32,33]. The general composition of UV-curable nanocomposites consists of silane-treated nanofillers dispersed in acrylic matrices [34,35]. In coatings, these matrices are usually a mixture of oligomers with an ester, epoxy or polyurethane core structure and reactive diluents [36]. For matrices in inkjet printing, the concentration of oligomers is reduced due to the viscosity limitation. Instead, mono- and difunctional acrylates are often added to the composition as diluents, and tri- and tetra-functional acrylates are added to increase the reaction speed [18,37]. Silica [29,35], alumina [29], clay [38,39], boehmite [40] and nanotitania [41] have been investigated as fillers and all have been shown to improve hardness and wear resistance in UV-curable materials. The nanoparticles increase the interaction density between the polymer chains by either covalently binding or by attaching themselves via van der Waals forces [28,35]. Silane coatings mediate the interaction. Commonly used molecules are 3-(trimethoxysilyl)propyl methacrylate and vinyltrimethoxysilane, which create bonds with the acrylic matrix. Non-covalently binding coatings such as trimethyl(phenyl)silane (TPS) also improve the scratch and abrasion resistance of nanocomposites [42]. The coating matches the surface energy of the small particles with the surface energy of the organic matrix and provides steric stabilization [43,44,45]. In addition to silanes, amphiphilic small molecules are often used which physically adsorb onto the ceramic surface [46]. Like TPS, they interact with the matrix based on van der Waals forces.

Particle-filled materials can be deposited using the inkjet printing process [47,48,49]. However, the influence of the ceramic contained in the material must be taken into account. The suspension can form a non-Newtonian behavior. In the case of shear thinning, low viscosity at high shear rates and high viscosity at low shear rates are observed [18]. This is important if the material is to reach the print head via supply channels where low shear rates prevail. When rheology ensures flow, dispensing through the printhead where high shear rates occur is usually possible. When printing the dispersion, the ejected material shows a tail, the ligament, which under certain parameters can form a drop [50]. The ligament, however, can also divide into one large and several small satellite droplets. Because of the different speed of the droplets, they hit the substrate at different places. The parameters that influence the ligament formation, as well as its splitting, are on the one hand the surface tension, the rheology and the density of the material and on the other hand the length and the height of the voltage pulse, which drives the piezoelectric membranes in the print head [51,52]. The viscosity of the material influences the weight, volume and speed of the drop. These values decrease as the viscosity increases. The forces acting in the print head cause the suspended ceramic to interact more strongly with each other. This can lead to agglomeration of the otherwise stable particles and to clogging of the nozzles [23,53]. To avoid this, the largest ceramic structures in the dispersion must not be larger than one-tenth of the nozzle diameter [18,23]. Furthermore, the tendency to agglomeration increases with increasing ceramic content [18,53].

The goal of this work was to investigate a simple and scalable process for the preparation of a 3D inkjet printable and ceramic-filled composite material for the production of components with improved tribological properties.

## 2. Materials and Methods

### 2.1. Materials

**Figure 1:**
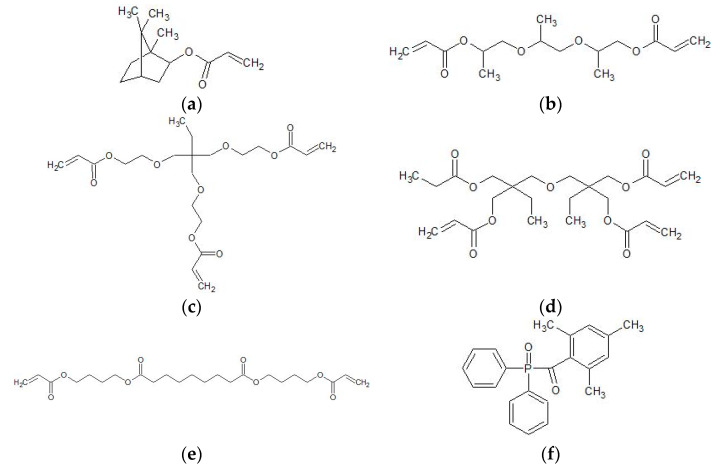
Components of the ultraviolet (UV)-curable matrix material: (**a**) isobornyl acrylate (IBOA), (**b**) tripropylene glycol diacrylate (TPGDA), (**c**) trimethylolpropane (EO) 3-trimethylolpropane triacrylate (TMPEO3TA), (**d**) di(trimethylolpropane)tetraacrylate (DTMPTA), (**e**) polyester acrylate genomer 3364, (**f**) diphenyl(2,4,6-trimethylbenzoyl)phosphine oxide (TPO). Genomer 3364 is a commercial product with a confidential chemical structure. The molecule shown is an example of a polyester acrylate.

The nano- and sub-micro alumina particles (TECNAN, Los Arcos, Navarra, Spain) were delivered in three average sizes: 13 nm, 35 nm and 100 nm. According to the manufacturer, the particle sizes were determined by calculation based on the specific surface area. The manufacturing method of the particles was the flame spray pyrolysis process. Before use, the ceramics were heat-treated, which is described in detail in the next chapter. Isopropyl alcohol (≥99.5% Carl Roth GmbH + Co KG, Karlsruhe, Germany), 2-[2-(2-methoxyethoxyethoxy)ethoxy] acetic acid (TODA, 96%, Sigma Aldrich, Darmstadt, Germany) were used as obtained. The UV-curable matrix material consisted of isobornyl acrylate (IBOA, 91.1%, Rahn AG, Zurich, Switzerland), tripropylene glycol diacrylate (TPGDA, 84.5%, Arkema, Colombes, France), trimethylolpropane (EO) 3-triacrylate (TMPEO3TA, 99.9%, KPX green chemicals, Seosan, South Korea), di(trimethylolpropane) tetraacrylate (DTMPTA, >969 %, Sigma Aldrich, Darmstadt, Germany), genomer 3364 (Rahn Chemicals, Zurich, Switzerland), and the photoinitiator diphenyl (2,4,6-trimethylbenzoyl) phosphine oxide (TPO, >98%, TCI, Eschborn, Germany) (Figure 1). All matrix components were used as obtained.

The composition of the matrix was determined on the basis of the formulation examples of Ref. [18], consisting of 45.7 wt.% of the monoacrylate IBOA, which after polymerization has a high glass transition temperature (Tg) and high toughness. The di- and triacrylates TPGDA and TMPEO3TA are contained in 26.5 wt.% and 10.9 wt.% respectively. Both are flexible crosslinkers, the latter having a better curing rate at higher viscosity. DTMPTA is a highly viscous tetrafunctional monomer with excellent reactivity. Its content is 3.5 wt.%. Genomer 3364 is a fast curing difunctional oligomer with a relatively low viscosity and good toughness. Its content in the matrix is 12.0 wt.%. TPO is a frequently used photoinitiator. Its content is 1.4 wt.% [18,37].

### 2.2. Filler Preparation and Charakterization

The morphologies of the ceramic fillers in the as-delivered condition were investigated by means of high-resolution scanning electron microscopy (Nova NanoSEM with EDAX EDX, FEI, Hillsboro, OR, USA) using the STEM mode. For the preparation, each of the three types of the recorded particles was suspended in ethanol, diluted and re-suspended in ethanol until a calculated concentration of 100 µg/mL was reached. Then a drop of each dispersion was placed on a carbon-coated TEM grid and dried for 12 h.

The specific surface area measurements were performed with an instrument from Micromeritics (Gemini VII 2390, Micromeritics GmbH, Unterschleißheim, Germany). The specific surface was calculated according to the standard Brunauer-Emmett-Teller method (BET). The samples were degassed in a glass tube under a vacuum. Nitrogen at −196 °C was then used as adsorbate for the determination of the adsorption/desorption isotherms.

**Figure A1:**
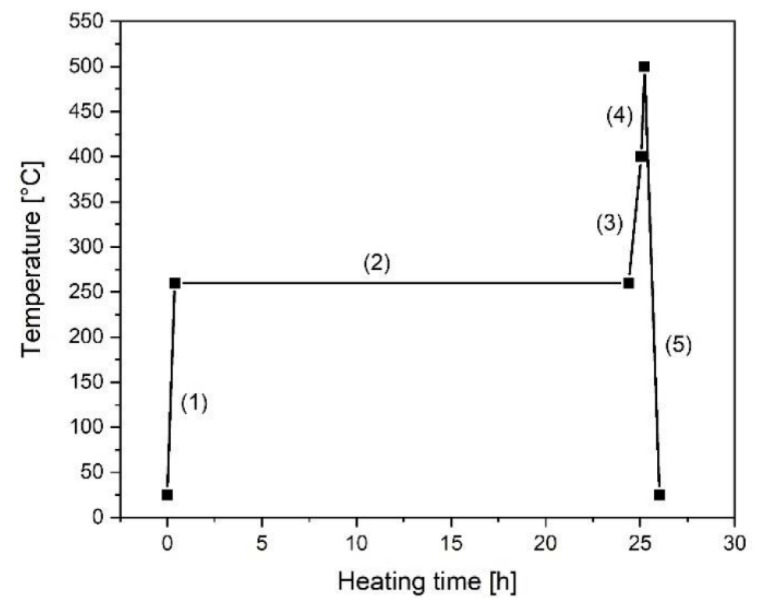
Temperature treatment program for the 35 nm-sized Al_2_O_3_ nanoparticles. The particles were heated up to 260 °C with a heating rate of 10 K/min (1) and the temperature was maintained for 24 h (2). Then the temperature was increased to 400 °C with a heating rate of 5 K/min (3) and to 500 °C with 10 K/min (4). The cooling rate to room temperature was 10 K/min (5)

Before use, the 13 nm and 100 nm particles were heated to 200 °C for 24 h to remove the physisorbed water from the surface and to improve the deposition of TODA. Due to different crystallography of the surface, the treatment of the 35 nm particles is more extensive. It is shown in Figure A1 and involves heating to 260 °C at a heating rate of 10 K/min (1), holding the temperature for 24 h (2), further heating to 400 °C at a heating rate of 5 K/min (3), then to 500 °C at 10 K/min (4), and cooling to room temperature at a cooling rate of 10 K/min (5).

The heat-treated particles were ground in a ball mill (PM400, Retsch GmbH, Haan, Germany). As equipment, a 125 mL grinding beaker with yttrium stabilized ZrO_2_ inner cladding and grinding balls of the same material, with a diameter of 2 mm, was used. The weight ratio of grinding balls to ceramic particles was 10:1 and the mechanical treatment time was 8 h. For the abrasion of the 13 nm fillers 11 g of the powder, 58 mL isopropyl alcohol and 110 g ZrO_2_ grinding balls were used. The process parameters for the 35 nm and 100 nm particles were the same. For both, 20 g of powder was ground with 60 mL isopropyl alcohol and 200 g ZrO_2_ grinding balls.

For the analysis of the particle size distribution, subsamples with a weight of 0.1 g were taken from all three suspensions after 5 min, 1 h and 8 h of grinding. The subsamples were further dispersed in 5 g isopropyl alcohol by vigorous shaking in vials. The analytical method was polarization differential scattering (Beckmann Coulter LS-230, Beckmann-Coulter Inc., Brea, CA, USA). Each measurement was performed by recording the background, inserting each subsample dropwise into the instrument and starting the measurement.

After 8 h of grinding, 2.8 g, 1.4 g and 0.4 g of the surfactant TODA were added to the suspensions of the 13 nm, 35 nm and 100 nm fillers, respectively. The mechanical treatment was continued for another 15 min. Afterwards, the samples were removed from the beakers and dried in a rotary evaporator for 1 h at 175 mbar and 50 °C. The amount of TODA corresponds to 2 mg/m^2^ of the particle surface. The obtained, heated and TODA-coated fillers were thermogravimetrically analyzed (STA 409C, Netzsch Group, Selb, Germany) in air atmosphere up to 1200 °C at a heating rate of 10 K/min.

### 2.3. Ink Preparation and Characterization

Each type of TODA-coated particles was prepared in the acrylate mixture described in Figure 1 at a nominal concentration of 1.0 vol%, 1.5 vol% and 2.0 vol%. An overview of the prepared samples is shown in Table 1. Resuspension was performed with a dispersing device(T 10 basic Ultra-Turrax, IKA GmbH, Staufen, Germany) at 14,450 rpm for 5 min and an ultrasonicbath (Sonorex Super RK103H, Bandelin electronic GmbH and Co. KG, Berlin, Germany) with a power of 560 W for 15 min. Before further characterization, the inks were filtered with a polytetrafluoroethylene (PTFE) filter with a pore diameter of 5 µm (Rotilabo^®^, Carl Roth, Darmstadt, Germany).

The thus prepared and filtered suspensions were analyzed thermogravimetrically in an air atmosphere of up to 1200 °C and a heating rate of 10 K/min. The dynamic viscosity of the filtered inks was determined using an automated dynamic shear rheometer (CVO 50, Malvern Instruments, Malvern, UK) with a cone-plate design, a cone diameter of 60 mm and an angle of inclination of 2°. The inks were measured at a constant shear rate of 500 s^−1^ and at a temperature between 20 °C and 60 °C and at a constant temperature of 60 °C and a shear rate variation between 1 s^−1^ and 500 s^−1^. The evaluation of the surface tension was performed with a drop shape analyzer (DSA 100, Krüss GmbH, Hamburg, Germany).

A Dimatix Materials Printer (DMP-2850, Fujifilm Dimatix Inc., Santa Clara, CA, USA) was used to investigate the jet properties of the materials. For the samples Unmodified, 13 III, 35 III and 100 III the drop shape, velocity, weight and volume were measured using the drop view of the printer. In the drop view, the drop behavior of the samples was observed over 10 min.

### 2.4. Composite Preparation and Characterization

**Figure 2:**
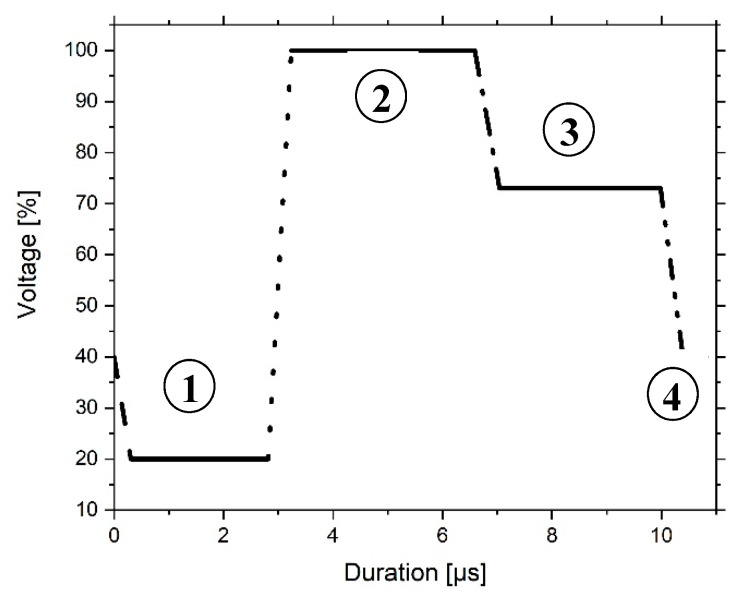
Waveform for the printing of the unmodified and ceramic-filled inks with four subdivided phases.

For the characterization of the tensile properties, specimens were printed with the Dimatix Materials Printer DMP-2850 according to DIN EN ISO 527-2 type A5 standard [54]. As printhead DMC-11610 cartridges were used with a drop volume of about 10 pL. The firing voltage was 30 V and the waveform was established according to Figure 2. It describes in four phases the bending of the piezo-electric actor in the printhead and the formation of the droplet. In the beginning, the firing voltage drops from 40% to 20% at a slew rate of 0.66. In 2.8 µs the ink is pumped into the firing chamber by the piezo-electric membrane. In the second phase, which is lasting for 3.8 µs, the curve rises to 100% at a slew rate of 1.90. Thereby, the ink is pushed out of the nozzle. During the third phase, the curve drops from 100% to 73% at a slew rate of 0.60 for 3.4 µs in order to prevent air suction. In the fourth phase, with a duration of 0.8 µs, the curve goes back to 40% from 73% with a slew rate of 0.80 and the pendant drop separates from the nozzle.

Five samples were printed simultaneously into a polydimethylsiloxane (PDMS) mold. After five printed layers each, the substrate was removed from the printer and irradiated with a UV light source (LED Spot 100 IC/HP IC, Dr. Hönle AG, Gräfelfing, Germany) at a wavelength of 405 nm and specific power of 540 mW/cm^2^ in the ambient atmosphere until the form was completely filled. An overview of the print samples is shown in Table 1. For the complete filling of the form 71, 58, 60, 76, 61, 73, 80, 150, 180 and 107 layers were applied for the samples unmodified, 13 I, 13 II, 13 III, 35 I, 35 II, 35 III, 100 I, 100 II and 100 III, respectively. The sample cross-section was measured three times with a caliper gauge according to the standard DIN EN ISO 527-2 type A5 in the longitudinal section of the sample. The tensile tests were performed on an universal testing machine (Zwick Z010, ZwickRoell GmbH & Co. KG, Ulm, Germany). Five specimens were tested per sample. The test was performed with a preload of 10 N at a strain rate of 1 mm/min, using a load cell with 2500 N to measure the resulting stress.

To assess the ceramic content of the tensile specimens after compression, a thermogravimetric analysis (TGA) was performed up to a temperature of 900 °C at a heating rate of 10 K/min. For each sample, three subsamples were taken from the entire cross-section of the connecting segment (length section). In addition, for samples 13 III, 35 III and 100 III subsamples were taken from the cross-section of the grip and from the lower and upper part of the grip to assess the heterogeneity of the ceramic content within a sample.

The glass transition temperature was measured via differential scanning calorimetry (Netzsch Group, DSC 204 F1 Phoenix, Netzsch Group, Selb, Germany). The temperature range was adjusted between −80 °C and 200 °C with a constant heating rate of 10 K/min. Three subsamples per sample were tested and analyzed with the instrument’s thermal analysis software.

## 3. Results and Discussion

### 3.1. Filler Characterization

**Figure 3:**
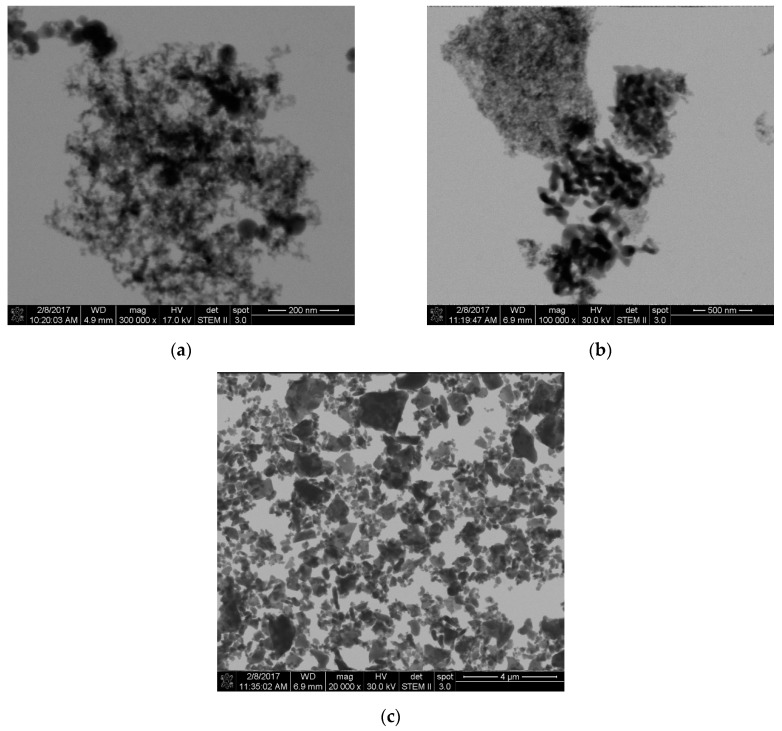
HRSEM images of as-received 13 nm (**a**), 35 nm (**b**) and 100 nm (**c**) sized alumina.

The examination of the ceramics in the as-received condition by high-resolution scanning electron microscopy (HRSEM) has shown the typical picture of materials produced by combustion (Figure 3). The 13 nm large powder consisted of spherical primary particles, which were connected by sintering necks and form fractal-like aggregates. The 35 nm-sized fillers show a bimodal composition of the primary particles. One cohort was in the 15 nm range and the other in the 50 nm range. The 100 nm-sized particles showed a morphology with a higher aspect ratio. The longitudinal diameter reached values up to 400 nm and exceeded the size specifications derived from the measurement of the specific surface. The BET values were 119 m^2^/g, 29 m^2^/g and 9 m^2^/g for the 13 nm, 35 nm and 100 nm particles, respectively.

**Figure 4:**
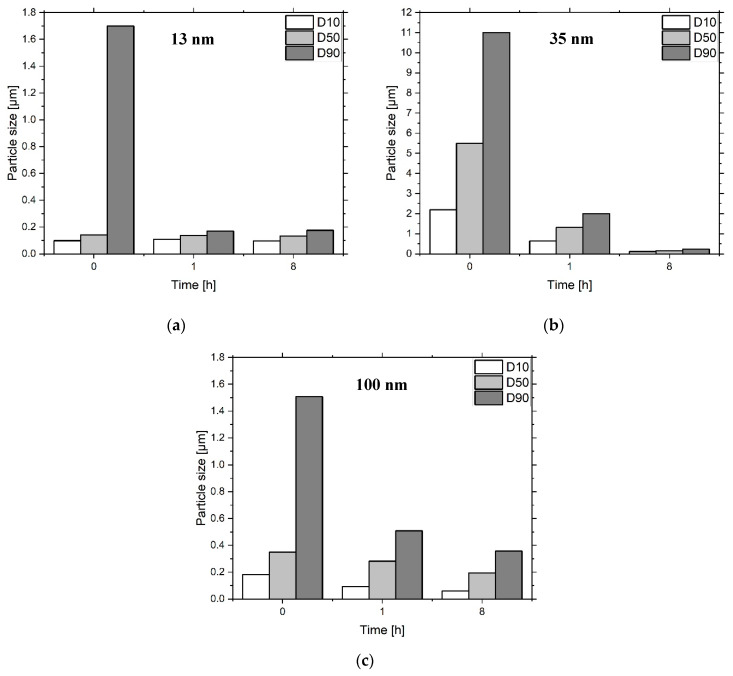
Particle size reduction in the ball mill for the 13 nm (**a**), 35 nm (**b**) and 100 nm (**c**) sized particles over a timeframe of 8 h.

The grinding of the heat-treated particles in the ball mill have shown the attrition of the aggregates and agglomerates. After an initial strong reduction, the values for D_10_, D_50_ and D_90_ remained relatively constant (Figure 4). The 13 nm particles showed no significant comminution after one hour, while the 35 nm and 100 nm particles showed the lowest values after 8 h. At this stage, the 100 nm powder was largely separated into its primary particles. The 13 nm and 35 nm-sized ceramics were still made of agglomerates.

**Figure 5:**
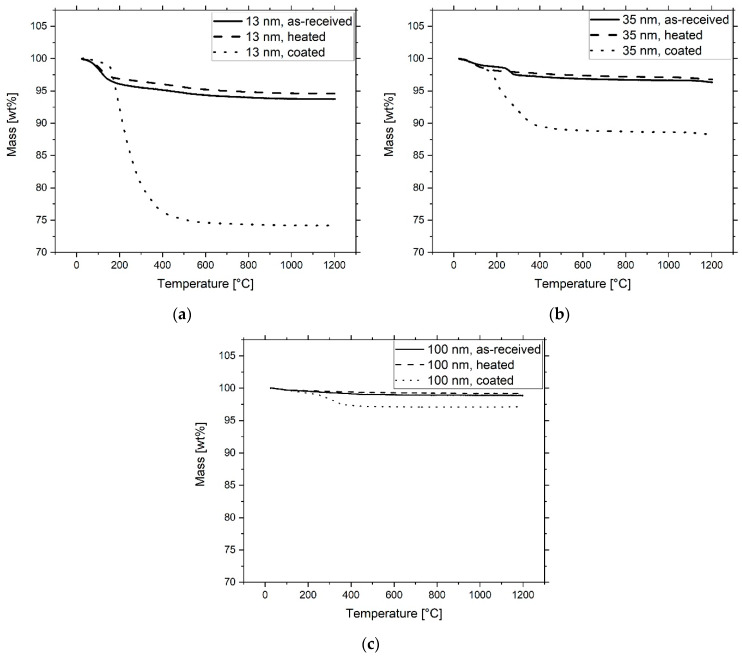
Thermogravimetric investigation of 13 nm (**a**), 35 nm (**b**) and 100 nm (**c**) as-received, heated and TODA coated filler particles.

All particles in the as-received state exhibited a mass loss in the TGA (Figure 5, Table 2). In the range of 20–200 °C, physically-bound water molecules were removed. Between 200–1200 °C, dehydration of the surfaces took place, reducing the concentration of chemically bound water in the form of OH groups. This can be seen from the difference between the curves of absorbed and heated particles. Physically bound water, however, was again deposited on the particle surfaces, as strong weight reduction below 200 °C was present for both types of curves. The 13 nm filler can be attributed to γ-Al_2_O_3_, which, under the given reaction conditions, was the thermodynamically more stable crystallographic form for fumed nano-alumina [55,56]. The 100 nm-sized particles were regarded as α-Al_2_O_3_. In the case of as-received 35 nm-sized particles, the curve which can be correlated with results in [57,58] indicated the presence of an Al(OH)_3_, e.g., bayerite, which was transformed into η-Al_2_O_3_ at 230 °C and to α-Al_2_O_3_ at 1200 °C [59,60]. The TGA measurement of the heated 35 nm powder indicates that a transformation of the Al(OH)_3_ components into η-Al_2_O_3_ has already taken place.

The ground and coated particles show a loss of 25.9 wt.%, 8.9 wt.% and 2.9 wt.% for fillers of 13nm, 35 nm and 100 nm size. Figure 5 shows desorption of TODA after 150 °C, which corresponds approximately to its boiling point. The complete removal of the molecules can be seen at about 400 °C. The curves show that the surfactant has displaced the physically adsorbed water from the particle surface, which has also been reported by other sources [61]. Therefore, the dispersant concentration, referred to as “measured TODA” in Table 2, is the difference between the weight reduction of coated and heated particles without the percentage of surface-bound moisture. The measured values for TODA are in good agreement with the amount of dispersant added during ball milling and are referred to as “weighted TODA” in Table 2. This result indicates that only limited evaporation occurred during the drying process of the powder.

### 3.2. Ink Characterization

**Figure 6:**
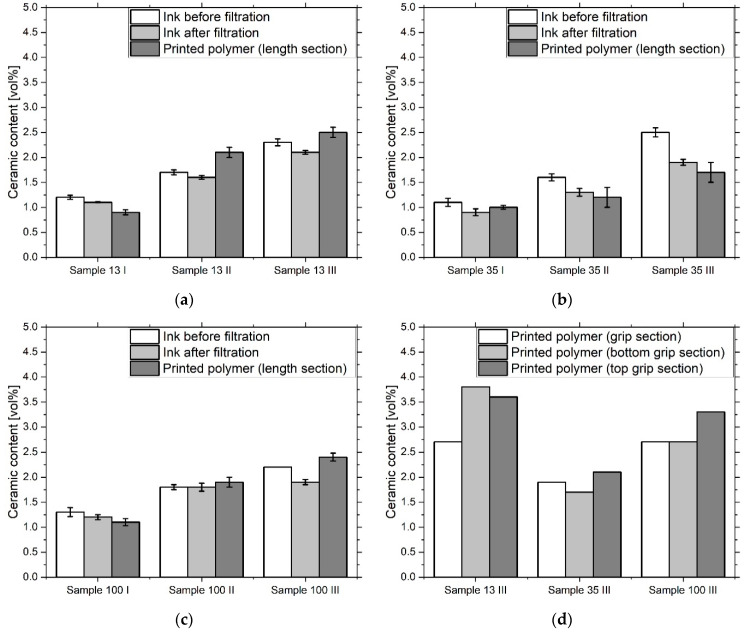
Ceramic content of the ink samples before and after filtration with a 5 µm filter and after printing and polymerization. The samples are 13 I, 13 II, 13 III (**a**), 35 I, 35 II, 35 III (**b**) and 100 I, 100 II, 100 III (**c**). The subsamples for the polymerized samples were taken in the longitudinal section of the tensile specimen. In (**d**) the ceramic content of samples 13 III, 35 III and 100 III is shown after the printing and polymerization. The subsamples were taken in the grip area, in the lower part of the grip and on top of the grip of the tensile specimen.

The inks had a higher ceramic content than the nominal value due to an excess of filler particles used in anticipation of losses during filtration (Figure 6). While filtration of the 13 nm inks worked well, three filters had to be used for the filled inks at 35 nm and 100 nm, as they occluded very quickly. The filled 13 nm and 100 nm inks show moderate ceramic residues in the filter. In contrast, the filled inks at 35 nm show a higher ceramic loss. The assumed η-Al_2_O_3_ surface of the former fillers, which differ from the other particles in their surface energy, should lead to increased agglomeration behavior.

**Figure A2:**
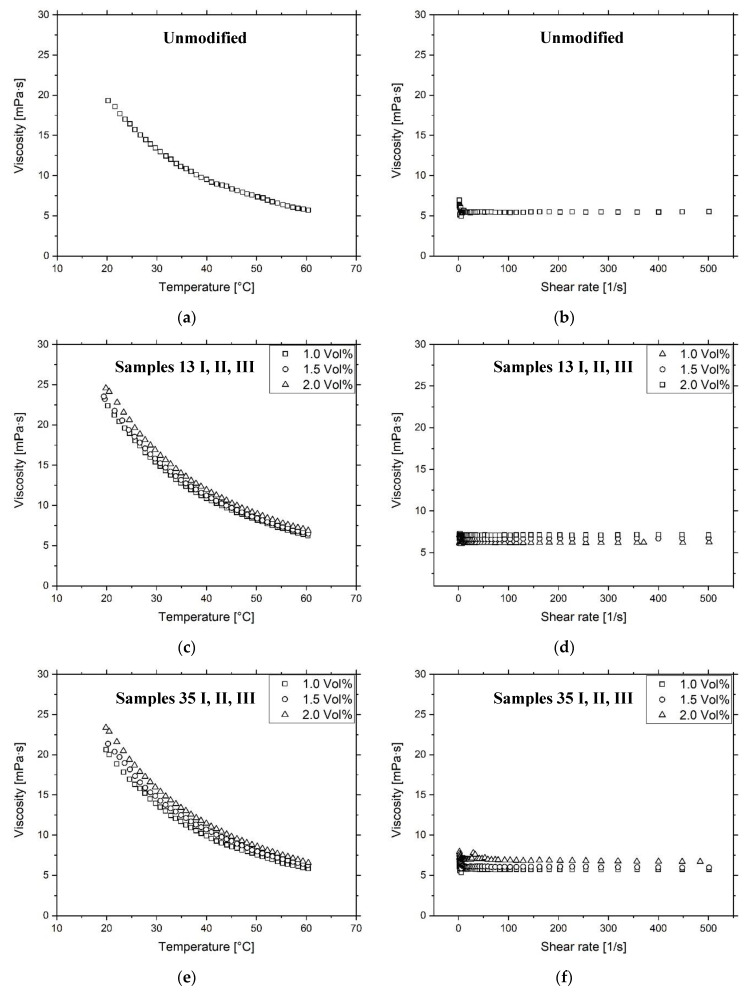

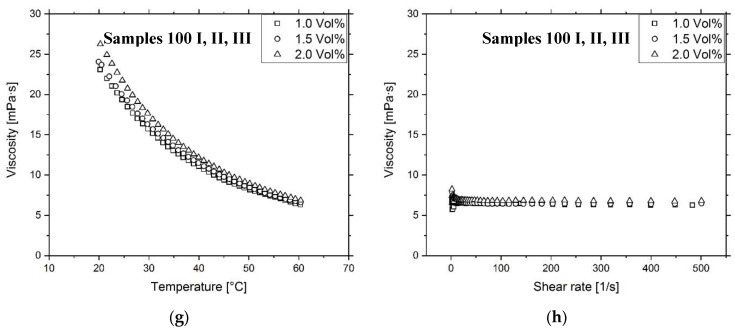
Rheology curves of the investigated inks in dependency of the temperature at a constant shear rate of 500 s^−1^ and in dependency of the shear rate at a constant temperature of 60 °C. The samples are Unmodified (**a**,**b**), 13 I, 13 II, 13 III (**c**,**d**), 35 I, 35 II, 35 III (**e**,**f**) and 100 I, 100 II, 100 III (**g**,**h**).

The filtered inks exhibit a slightly higher viscosity with each increase in particle concentration (Table A1, Figure A2). Due to their large surface area, the submicro- and nanoparticles immobilize the matrix and form network structures within the matrix [62]. Due to the low ceramic content, the suspensions exhibit Newtonian behavior, which is advantageous for the printing process [18]. The addition of fillers increases the surface tension of the printing ink (Table A1). Since the TODA limits the surface energy of the particles and the ceramic concentration is low, the growth is very moderate. The different surface areas of the powders have no influence on the results.

**Figure A3:**
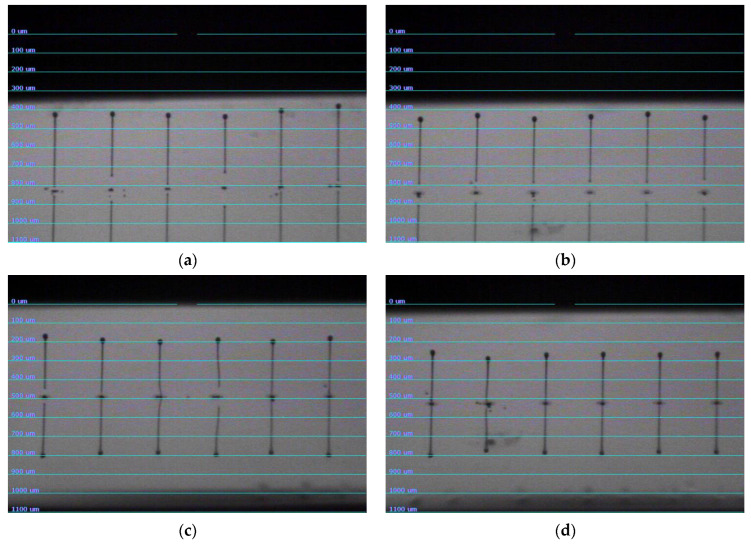
Ink jetting properties for the ink samples Unmodified (**a**), 13 III (**b**), 35 III (**c**) and 100 III (**d**). The inks show the formation of a ligament without the disintegration into satellite droplets.

The results of the jetting test are shown in Figure A3. The investigated samples show an elongated ligament. There is no visible formation of satellite droplets. The drop velocity is 11.5 m/s, 10 m/s, 9 m/s and 8 m/s for the samples Unmodified, 13 III, 35 III and 100 III, respectively (Table 3). The drop velocity is within the range expected for inkjet printing [18]. The initial drop volume and weight is on average 8.3 pL and 9 ng, respectively. Due to the low viscosity of the materials, the results are relatively similar. In samples 35 III and 100 III, a slight decrease in volume and weight can be seen, which is due to the ceramic content. A decrease in drop quality could not be observed after 10 min jetting time.

### 3.3. Composite Characterization

**Figure 7:**
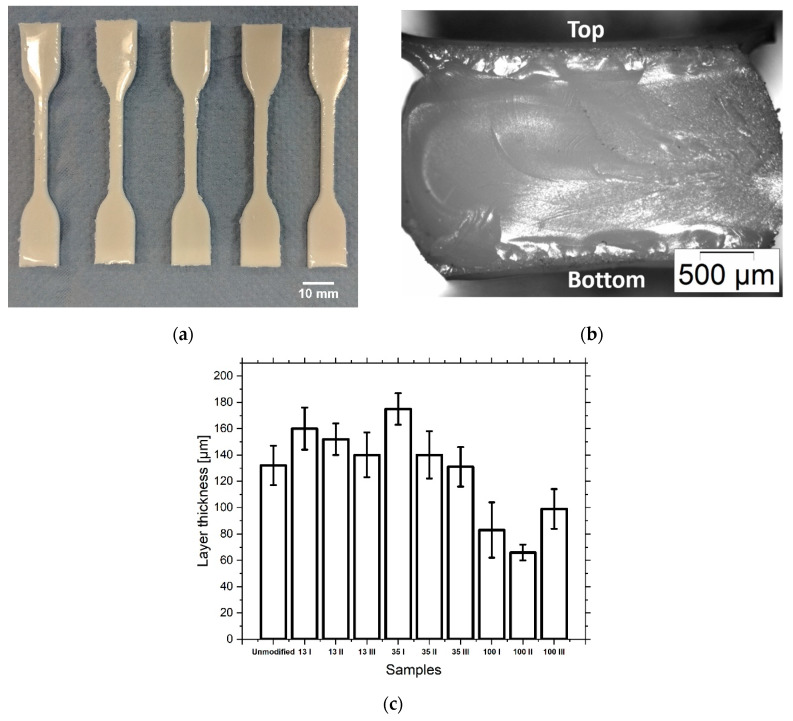
Printed tensile specimens of sample 100 III with 2 vol% fillers of 100 nm size (**a**). Cross-section of the specimen (**b**). The layer thickness of five subsequently printed layers with a UV curing step after their deposition for all printed samples (**c**).

The result of the tensile test specimens printing is shown for sample 100 III (Figure 7a). The cross-section of the sample is shown in Figure 7b. The printing into the PDMS form gives the top of the specimen a concave curvature and the sides are rounded.

Examination of the ceramic content of the tensile specimens after printing showed that the composition of the material changed during the printing process (Figure 6). While the ceramic content of samples 13 I and 13 II decreased slightly, sample 13 III showed a significant increase. In the samples filled with 35 nm, the ceramic content showed only slight deviations. With 100 nm filled samples, only 100 III showed a significant increase in the ceramic content. Although no investigations have yet been carried out to precisely answer the question of why the deviation of the ceramic content is so strong in certain samples, two influencing factors can be named. On the one hand, agglomeration and occlusion of the print head nozzles can be the cause of a reduction in the ceramic content. However, sedimentation within the print head can increase the ceramic content by delivering a more concentrated suspension. Looking at the thickness of the cured layers (Figure 7c), it is noticeable that it is higher in unmodified, 13 nm and 35 nm filled samples than in 100 nm filled samples.

The reason for this is the partial occlusion of the nozzle, which leads to a reduced droplet volume. However, as was observed during the filtration of the inks, the ceramic content of the suspensions filled with 100 nm particles was not reduced by the partially occluded nozzle.

It is also shown that the distribution of the ceramic varies within a sample (Figure 6d). The concentration was measured in the grip and the middle segment (length section) of the samples. The samples taken from the cross-sections show few differences. Subsamples of 13 III and partly of 100 III, taken from the top and bottom of the samples, vary in their ceramic concentrations relative to each other and slightly more to the subsamples from the cross-sections. The explanation for this occurrence could be the preferred accumulation of the ceramic on the PDMS mold wall. The increased interaction with this interface is particularly evident in the concave shape of the upper side of the sample.

The glass transition temperature for the unfilled resin is 66 ± 7 °C (Table A2). The addition of ceramics does not lead to any significant changes in the glass transition temperature due to the low ceramic content.

**Figure 8:**
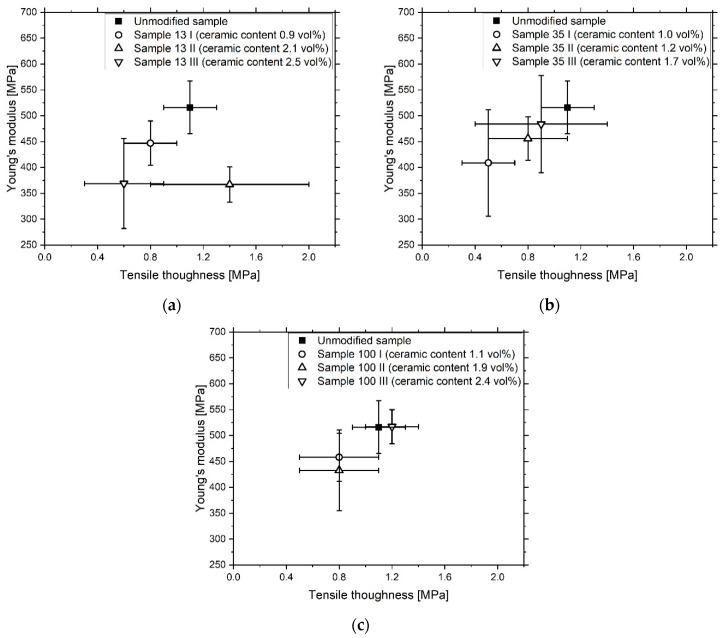
Stiffness/tensile toughness relationship of the printed 13 nm (**a**), 35 nm (**b**) and 100 nm (**c**) filled samples with varying ceramic content.

**Figure A4:**
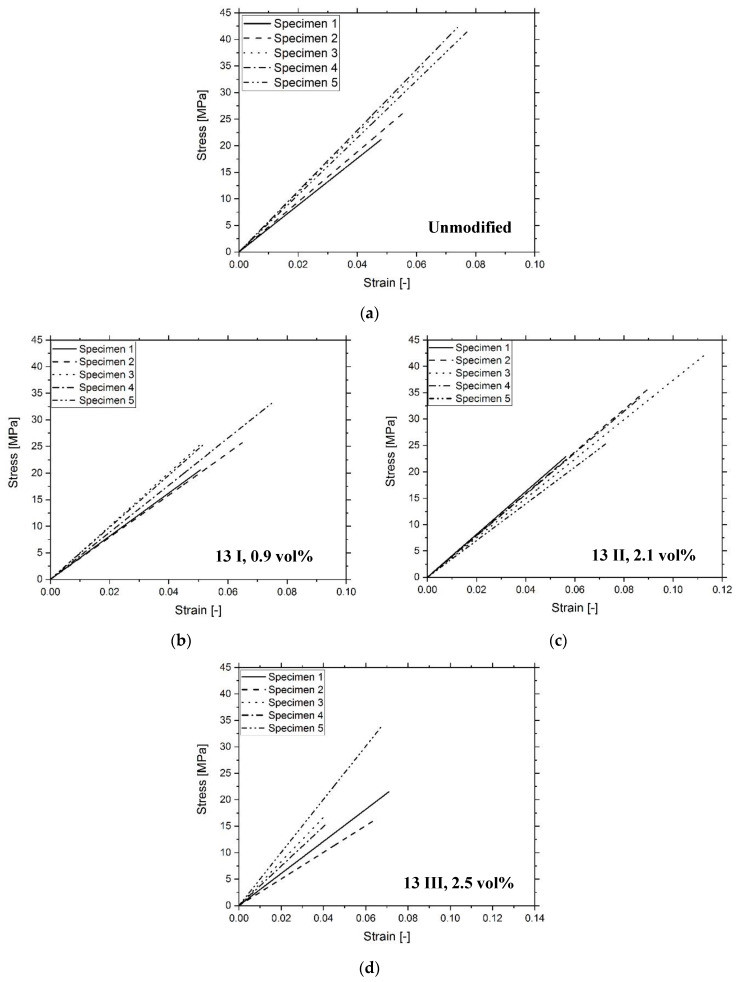

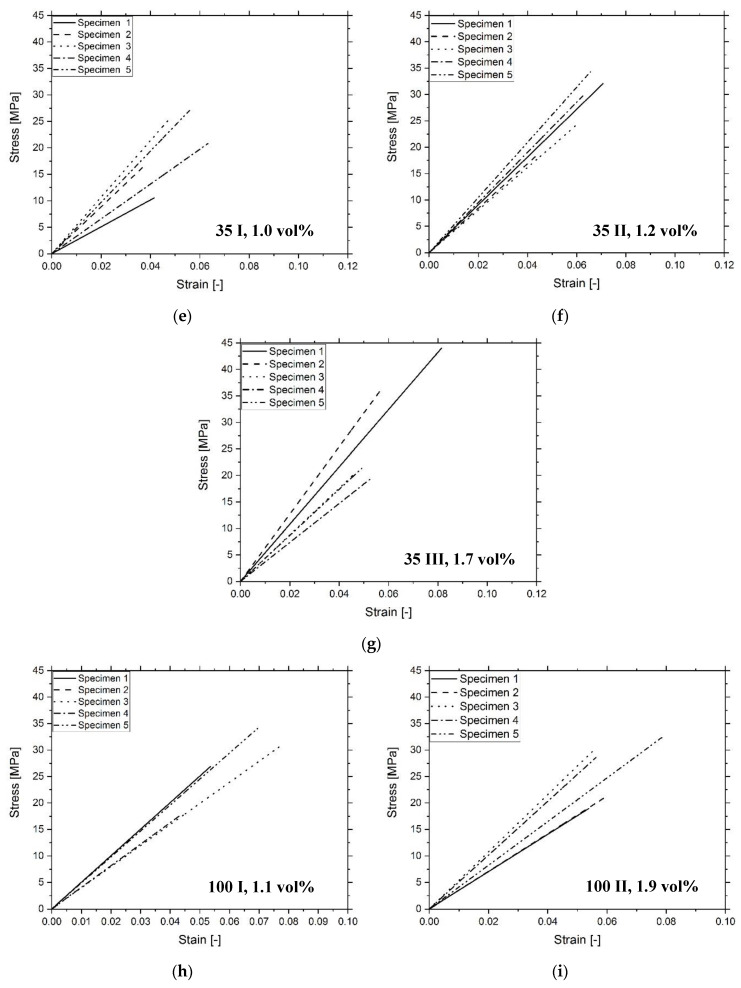

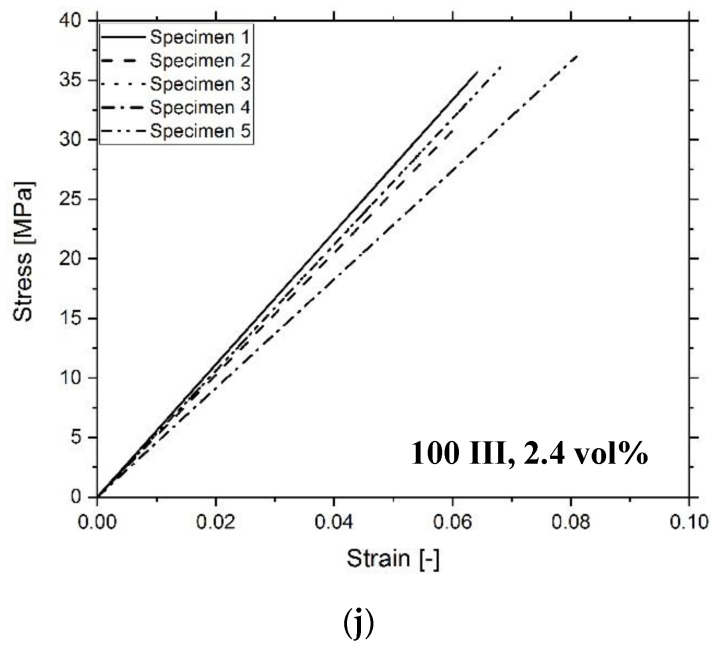
Stress-strain curves of unmodified (**a**), 13 nm ceramic modified (**b**–**d**), 35 nm ceramic modified (**e**–**g**), 100 nm ceramic modified (**h**–**j**) photopolymers. The filler content for each of the three particle sizes is shown in the diagrams.

The Young’s modulus of elasticity of the unfilled resin shows the second-best result at 516 ± 51 MPa (Figure 8, Figure A4, Table A2). The elastic moduli of the other samples, with the exception of 100 III, show lower values, which could indicate the scattering effect of the fillers and the associated reduction in the degree of crosslinking of the polymer matrix [28]. Sample 100 III exhibits the highest stiffness at 517 ± 33 MPa. Compared to the filled samples with the 13 nm and 35 nm particles, sample 100 III shows a thinner layer thickness, which could reduce the influence of UV light scattering. The tensile strength and toughness of the samples are subjected to minor fluctuations. However, in accordance with the results of the stiffness measurement, the filled samples show slightly lower values, with the exception of 13 II and 100 III.

## 4. Conclusions

The size of the particles produced by the flame spray pyrolysis was adjusted during grinding in the ball mill. It could be shown that the prior removal of physisorbed water on the particle surface is not necessary, as it is displaced by TODA. Furthermore, TODA does not evaporate from the particle surface during the drying of the ceramic in the rotary evaporator. The dispersion of the coated nanoparticles in the organic acrylate matrix was mediated by TODA and leads to a low-viscosity suspension with Newtonian behavior. The filterability of the three investigated particle sizes differs due to the different particle size and surface chemistry. The 35 nm particles are the least stable due to the η-Al_2_O_3_ surface. The printing properties of the filled inks differ only slightly from those of the unfilled inks. The ceramic content of the printed composites shows limited changes after the printing process, which is due to sedimentation in the print head. The 100 nm nanoparticles can partially clog the print head and lead to lower deposition rates. Switching to smaller particle sizes would be one solution. Light scattering by the fillers can influence the cross-linking and thus the mechanical properties of the material. Print layers in the range of 100 µm have shown better results. The aim of this work was initially to develop a simple, scalable process for the production of a 3D inkjet printable and ceramic-filled composite material that can be used to produce abrasion-resistant components with adapted tribology. This goal was achieved when it comes to the production of materials with a low ceramic content of up to 2 vol%. The scope of the preparation steps include grinding, physical coating and drying of the particles. Resuspension in the photopolymer can be carried out with simple laboratory equipment before printing. The stability of the material allows inkjet printing and does not significantly deteriorate the mechanical properties of the photopolymer if the production parameters are correctly adjusted.

## Figures and Tables

**Table 1:** Overview of the samples which describe the liquid inks as well as the solidified specimens. The Al_2_O_3_ fillers are coated with TODA.

Sample	Al_2_O_3_ Particles	Ceramic n. ^1^ (vol%)
Unmodified	-	0.0
13 I	13 nm coated	1.0
13 II		1.5
13 III		2.0
35 I	35 nm coated	1.0
35 II		1.5
35 III		2.0
100 I	100 nm coated	1.0
100 II		1.5
100 III		2.0

^1^: nominal.

**Table 2:** Maximum mass loss of as-received, heated and coated fillers during thermogravimetric analysis. The weighted TODA concentration, i.e., the amount of dispersant added during ball milling, is related to the TODA concentration measured in the TGA.

Al_2_O_3_ Nanoparticles	Mass Loss (wt.%)	Weighted TODA (wt.%)	Measured TODA (wt.%)
13 nm as received	6.3	-	-
13 nm heated	5.4	-	-
13 nm coated	25.9	23.6	23.5
35 nm as received	3.7	-	-
35 nm heated	3.2	-	-
35 nm coated	8.9	7.1	7.6
100 nm as received	1.2	-	-
100 nm heated	0.8	-	-
100 nm coated	2.9	2.0	2.4

**Table 3:** Ink jetting properties for the ink samples Unmodified (a), 13 III (b), 35 III (c) and 100 III (d).

Samples	Drop Velocity (m/s)	Drop Volume (pL)	Drop Weight (ng)	Jetting Time (s)
Unmodified	11.5	7.8	8	>600
13 III	10	10	11	>600
35 III	9	8.2	9	>600
100 III	8	7.3	8	>600

## Appendix A

**Table A1:** The viscosity of the samples after filtration at 60 °C and a shear rate of 500 s^−1^. The surface tension of the inks after filtration.

Sample	Viscosity (60 °C) (mPa∙s)	Surface Tension (mN/m)
Unmodified	5.5 ± 0.2	27.3 ± 1.5
13 I	6.2 ± 0.02	30.0 ± 2.4
13 II	6.7 ± 0.08	32.5 ± 2.1
13 III	7.1 ± 0.11	30.3 ± 2.4
35 I	5.7 ± 0.19	27.2 ± 1.3
35 II	5.9 ± 0.04	32.0 ± 2.4
35 III	6.7 ± 0.13	29.7 ± 2.3
100 I	6.3 ± 0.12	30.0 ± 2.9
100 II	6.4 ± 0.04	30.4 ± 1.9
100 III	6.8 ± 0.06	31.9 ± 1.2

**Table A2:** Characteristics of the tensile samples after printing: Glass transition, Young’s modulus, ultimate tensile strength and tensile toughness.

Sample	T_g_ (°C)	E (MPa)	σ_max_ (MPa)	U_T_ (MPa)
Unmodified	66 ± 7	516 ± 51	33 ± 6	1.1 ± 0.2
13 I	59 ± 4	447 ± 43	26 ± 4	0.8 ± 0.2
13 II	58 ± 4	367 ± 34	32 ± 7	1.4 ± 0.6
13 III	55 ± 5	369 ± 87	21 ± 7	0.6 ± 0.3
35 I	64 ± 8	409 ± 103	20 ± 6	0.5 ± 0.2
35 II	62 ± 1	456 ± 42	28 ± 6	0.8 ± 0.3
35 III	63 ± 1	484 ± 94	28 ± 10	0.9 ± 0.5
100 I	66 ± 5	458 ± 46	27 ± 6	0.8 ± 0.3
100 II	64 ± 5	433 ± 78	29 ± 6	0.8 ± 0.3
100 III	66 ± 3	517 ± 33	35 ± 2	1.2 ± 0.2
